# Psoriasiform rash following maculopapular eruption during the continued administration of pembrolizumab^☆^

**DOI:** 10.1016/j.abd.2023.03.012

**Published:** 2024-06-29

**Authors:** Mayu Sato, Toshiyuki Yamamoto

**Affiliations:** Department of Dermatology, Fukushima Medical University, Fukushima, Japan

Dear Editor,

A 73-year-old male with unresectable head and neck squamous cell carcinoma was treated with monthly pembrolizumab (200 mg). He had no family or personal history of skin disease, including psoriasis. Soon after the second administration, he developed itchy erythema, which spread to the trunk and extremities. Physical examination showed coalesced erythema and papulaes on the trunk and extremities (Fig. 1). Histopathological examination showed vacuolar changes of the basement membrane of the epidermis and prominent infiltration of eosinophils in the upper dermis (Fig. 2). Because grade of cutaneous Immune-Related Adverse Events (irAEs) was 2, pembrolizumab was continued under treatment with topical corticosteroid; however, itchy erythema gradually worsened, and after 5 doses pembrolizumab was discontinued. Oral prednisolone was administered at a dose of 5‒10 mg/day, but the skin lesions further worsened and a different skin rash appeared. One month after discontinuing pembrolizumab, infiltrative erythematous and slightly keratotic plaques were observed on the trunk and extremities (Fig. 3). Mucous involvement was not observed. A second biopsy revealed subcorneal neutrophilic abscess, individual cell keratinization, liquefaction degeneration of the basement membrane region and dermal cellular infiltrates containing eosinophils (Fig. 4). After increasing the dose of oral prednisolone to 30 mg/day for 2 weeks, the skin lesions improved. After prednisone discontinuation, there was no recurrence of skin lesions.

**Figure 1:**
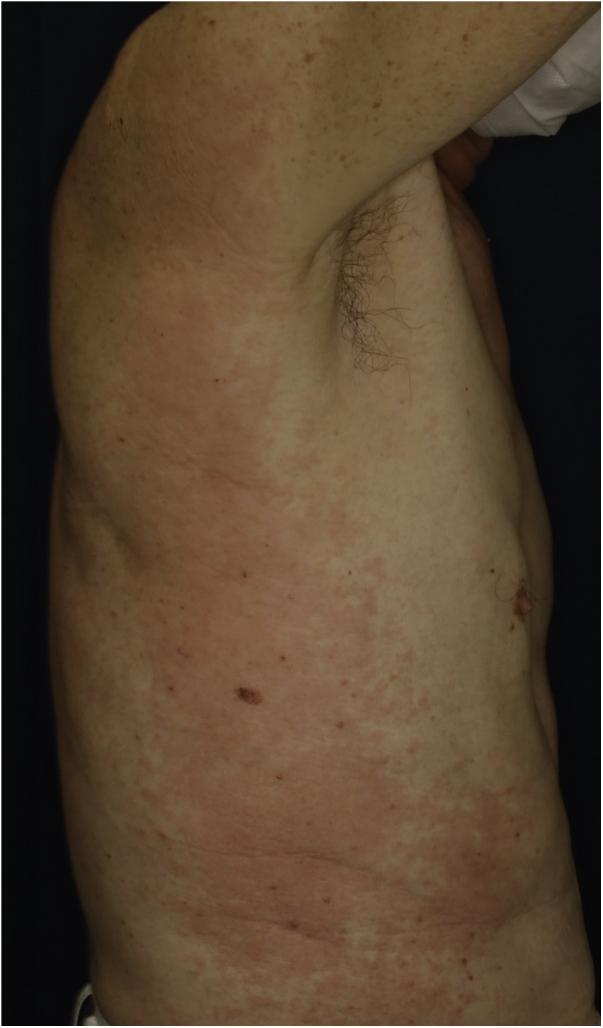
Diffuse coalescent erythema and paplular lesions on the trunk and extemities.

**Figure 2:**
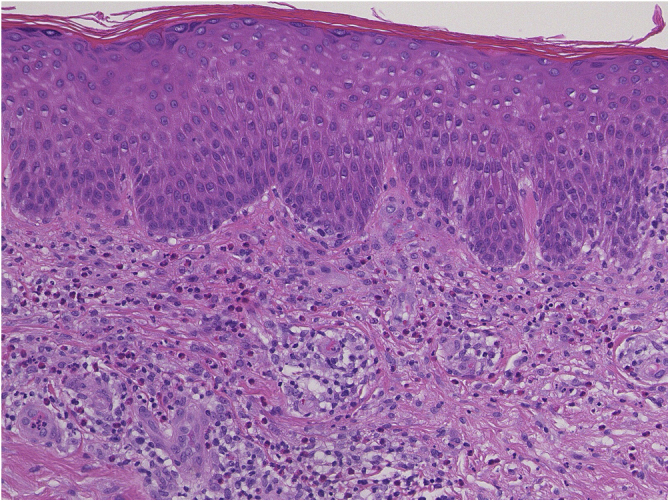
Histopathological features showing vacuolar degeneration of the basement membrane of the epidermis and cellular infiltration of mononuclear cells and eosinophils in the upper dermis.

**Figure 3:**
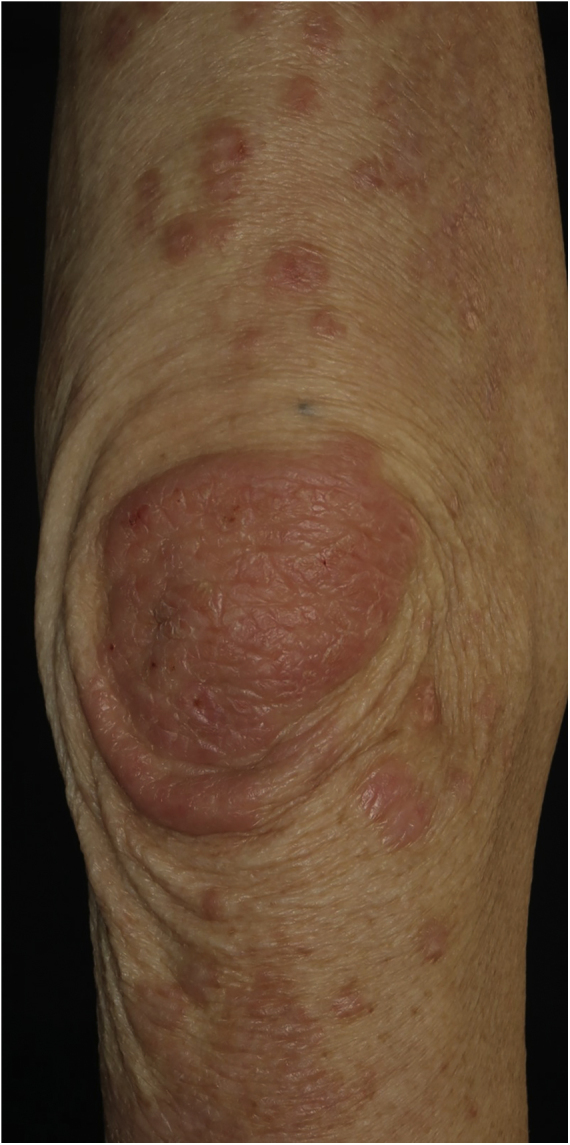
Well-defined keratotic erythema on the elbow.

**Figure 4:**
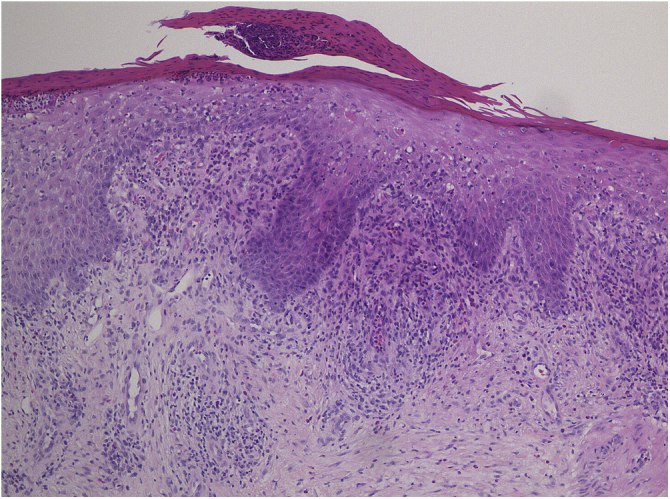
Histopathological features showing subcorneal microabcess, epidermal proliferation, scattered individual cell keratinization, and liquefaction degeneration in the basement membrane region.

The present case initially developed a maculopapular or morbiliform coalesced erythema on the trunk and extremities 5 weeks after initiation of pembrolizumab. Initial biopsy specimen showed interface changes of the epidermis and dermis junction, and prominent eosinophil infiltration in the upper dermis. Because pembrolizumab was effective for SCC, pembrolizumab was continued thereafter using topical corticosteroid, but itchy erythema was gradually worsened, and pembrolizumab was stopped after 5 doses. His rash was treated with oral prednisolone (initially 5 mg/day for 1 week, and 10 mg/day for another 1 week); however, 2 months later, his skin rash deteriorated, and clinical phenotype changed along with the gradual worsening of the skin rash. Of interest, the second biopsy revealed psoriasiform changes such as subcorneal neutrophilic abscess, while individual epidermal cell keratinization and liquefaction degeneration were also observed.

Although it is well-known that various cutaneous irAEs appeared by the use of immune checkpoint inhibitors (ICIs),^1^, ^2^, ^3^ there are few reports on phenotypic changes of cutaneous irAEs during long-term use of ICIs. To date, there is one report in which pembrolizumab caused LP-like lesions, which thereafter developed bullous pemphigoid.^4^ They speculated that ICIs evoked the breakdown of immune tolerance to the epidermal basement membrane zone through lichenoid reactions. In addition, resumption of ICIs may induce both the same and different cutaneous lesions,^5^ with an escalated grade. In the majority of cases, irAEs are grade 1 or 2, and can be controlled under topical treatment without discontinuation of ICIs. However, in the present case, cutaneous manifestation worsened during continued administration of pembrolizumab, and beyond the control of topical and oral corticosteroids. Pembrolizumab was finally discontinued, but skin lesions further deteriorated even after the discontinuance. Furthermore, the phenotype of skin lesions changed from initially coalesced erythema to keratotic plaques, with. Histopathological features such as psoriasiform changes. Thereafter, skin lesions gradually worsened and the intervals of pembrolizumab administration were prolonged or skipped; however, skin lesions were uncontrolled and finally pembrolizumab was discontinued and oral prednisolone was administered. But 2 months after discontinuation of pembrolizumab, different phenotypes of skin lesions appeared, which were proved to be psoriasiform by skin biopsy. Therefore, careful and long-term monitoring, as well as management of cutaneous irAEs are required.

## Financial support

None declared.

## Authors’ contributions

Mayu Sato: designed the study, performed the research and contributed to the analysis and interpretation of data, wrote the initial draft of the manuscript, read and approved the final version of the manuscript.

Toshiyuki Yamamoto: designed the study, assisted in the preparation of the manuscript, read and approved the final version of the manuscript.

## Conflicts of interest

None declared.
